# Enhancing Polylactic Acid Properties with Graphene Nanoplatelets and Carbon Black Nanoparticles: A Study of the Electrical and Mechanical Characterization of 3D-Printed and Injection-Molded Samples

**DOI:** 10.3390/polym16172449

**Published:** 2024-08-29

**Authors:** Salvador Giner-Grau, Carlos Lazaro-Hdez, Javier Pascual, Octavio Fenollar, Teodomiro Boronat

**Affiliations:** 1 Textile Industry Research Association (AITEX), Plaza Emilio Sala, 1, 03801 Alcoy, Spain; salgigr1@alumni.upv.es (S.G.-G.); jpascual@aitex.es (J.P.); 2 Instituto Universitario de Investigación de Tecnología de Materiales (IUITM), Universitat Politècnica de València (UPV), Plaza Ferrándiz y Carbonell 1, 03801 Alcoy, Spain; ocfegi@epsa.upv.es (O.F.); tboronat@dimm.upv.es (T.B.)

**Keywords:** PLA, nanoadditives, 3D printing, injection molding

## Abstract

This study investigates the enhancement of polylactic acid (PLA) properties through the incorporation of graphene nanoplatelets (GNPs) and carbon black (CB) for applications in 3D printing and injection molding. The research reveals that GNPs and CB improve the electrical conductivity of PLA, although conductivity remains within the insulating range, even with up to 10% wt of nanoadditives. Mechanical characterization shows that nanoparticle addition decreases tensile strength due to stress concentration effects, while dispersants like polyethylene glycol enhance ductility and flexibility. This study compares the properties of materials processed by injection molding and 3D printing, noting that injection molding yields isotropic properties, resulting in better mechanical properties. Thermal analysis indicates that GNPs and CB influence the crystallization behavior of PLA with small changes in the melting behavior. Dynamic Mechanical Thermal Analysis (DMTA) results show how the glass transition temperature and crystallization behavior fluctuate. Overall, the incorporation of nanoadditives into PLA holds potential for enhanced performance in specific applications, though achieving optimal conductivity, mechanical strength, and thermal properties requires careful optimization of nanoparticle type, concentration, and dispersion methods.

## 1. Introduction

Polylactic acid (PLA) is a biodegradable and biocompatible polymer derived from glucose-rich sources such as corn, sugar beets, and other sugar-rich crops. This biopolymer is gaining considerable attention in the industry due to its potential to reduce dependence on petroleum-based plastics and its lower environmental impact [1]. PLA is used in food packaging, textiles, medical devices, and 3D printing and is noted for its ability to biodegrade in industrial composting conditions and its biocompatibility [2,3]. Despite its many advantages, PLA has significant limitations in applications requiring electrical conductivity. To address these limitations, conductive additives such as graphene nanoplatelets (GNPs) and carbon black (CB) are incorporated [4]. These additives enhance PLA’s properties by forming a conductive network within the polymer matrix, facilitating the passage of electrical current. Besides improving conductivity, these additives increase PLA’s mechanical strength, thermal stability, and wear resistance, broadening its range of applications in flexible electronic components, sensors, and energy storage devices [5,6]. Conductivity in plastic materials is a growing area of interest due to the trend toward miniaturization and flexibility in modern technology. Conductive plastics combine the mechanical and processable properties of polymers with the ability to conduct electricity, opening new possibilities in the design and manufacture of electronic devices. This feature is especially valuable for applications such as flexible displays, smart clothing, and portable medical devices [7]. Conductive plastics are also essential in sensors and actuators because they can respond to external stimuli and convert them into electrical signals, making them ideal for real-time detection and monitoring applications. Additionally, these materials are crucial in the energy storage industry, as they are used in the manufacture of battery and supercapacitor components, offering a combination of light weight, flexibility, and efficient storage capacity [8]. GNPs are nanomaterials composed of stacked layers of graphene, providing high strength, rigidity, electrical and thermal conductivity, and a large surface area. They are typically supplied in fine powder form and can be dispersed in various solvents or polymer matrices [9,10]. CB is an amorphous form of carbon known for its high surface area and ability to enhance the electrical and mechanical properties of materials. It is also supplied in fine powder form and can be dispersed in polymer matrices or solvents [10,11].

Injection molding is a manufacturing process used to produce high-volume parts with high precision and consistency. This method allows the production of parts with complex geometries and precise tolerances, which is ideal for manufacturing large volumes of parts quickly and efficiently, ensuring uniform distribution of additives in the polymer matrix. However, it has drawbacks such as high initial cost, complexity in parameter control, and limitations in particle orientation [12,13]. Additive manufacturing, commonly known as 3D printing, creates three-dimensional objects by successively adding material layer by layer [14]. This method allows the creation of complex and customized geometries, uses only the necessary material, and facilitates rapid prototyping. However, it can be slower than injection molding, and 3D-printed parts can have inferior mechanical properties and require post-processing to improve surface finish [15,16]. Choosing between injection molding and additive manufacturing depends on the specific needs of the project, including production volume, design complexity, required material properties, and associated costs [17,18]. But some properties like the rheological behavior must be considered in order to employ the developed materials in the additive manufacturing process in order to achieve a proper behavior [19].

Additives like GNPs and CB form a network of conductive paths within the matrix, facilitating electron movement [20]. Good conductivity in composite materials ensures effective use in these applications, enhancing the performance and durability of final products [21]. Percolation theory explains how a global property, such as conductivity, emerges in a system composed of individual components that do not necessarily possess that property. In conductive composite materials, percolation refers to the process by which conductive particles form a continuous network within the polymer matrix, allowing the flow of electrical current. To achieve percolation, the concentration of conductive particles must reach a critical threshold known as the percolation threshold. Below this threshold, the particles are dispersed in isolation, and the material remains insulating. Once the threshold is exceeded, the particles form an interconnected network that enables conductivity throughout the composite [22]. The orientation and dispersion of conductive particles within the polymer matrix are crucial factors affecting the percolation process. The alignment of particles can significantly influence the formation of the conductive network. For instance, GNPs tend to align parallel during certain manufacturing processes, facilitating the formation of efficient conductive paths. Uniform dispersion of the particles is essential to achieve and exceed the percolation threshold, while poor dispersion can result in particle agglomerates that do not effectively contribute to overall properties [23]. Interactions between particles, such as attraction or repulsion, also affect the ability to form a conductive network. Manufacturing techniques and the choice of additives can help control these interactions to optimize conductivity [24,25]. The integration of carbon nanoparticles into polylactic acid (PLA) significantly enhances its mechanical, thermal, permeability, and aesthetic properties, greatly expanding its range of applications. These nanoparticles fortify the PLA matrix, boosting its strength and rigidity, which is essential for high-durability structural applications. The superior thermal conductivity of these nanoparticles also enhances heat distribution within PLA, elevating its thermal stability and suitability for more challenging conditions. In the packaging sector, adjusting PLA’s permeability barrier with nanoparticles helps preserve food longer by reducing gas permeability, particularly to oxygen. Furthermore, the aesthetic and optical enhancements provided by these nanoparticles enable customization of the material’s appearance, catering to specific needs such as decreased transparency or particular colors, thus broadening PLA’s applicability in diverse industries [26].

Numerous studies have investigated PLA-based composites with carbon-based fillers, particularly carbon nanotubes (CNTs) and carbon black (CB), to improve both electrical conductivity and mechanical properties. For instance, research by Petrényi et al. demonstrated that while the addition of CNTs alone to PLA could slightly enhance electrical conductivity, the effect was significantly amplified when combined with carbon fibers. This synergistic interaction facilitated the formation of conductive networks, achieving conductivities up to 0.355 S/cm. However, the study also highlights challenges such as increased brittleness and processing difficulties due to higher viscosity, which are common limitations in PLA composites incorporating conductive fillers [27].

Despite some advancements, achieving a balance between mechanical strength and electrical performance remains a challenge. When dispersion of the carbon-based fillers is not optimized, conductivity gains tend to be modest, and mechanical properties can suffer, often leading to brittle composites. Therefore, while there have been successes in enhancing conductivity, these gains often come at the cost of mechanical performance, especially when high filler content is required to reach the percolation threshold [27,28].

The injection molding process can cause GNPs and CB particles to align in the flow direction, resulting in anisotropic conductivity. However, high pressure and rapid solidification can make uniform particle dispersion challenging. Additive manufacturing allows greater control over the orientation and dispersion of additives, optimizing conductivity in different directions according to application needs and improving performance in practical applications [27,29,30]. Both injection molding and additive manufacturing significantly impact the orientation and dispersion of conductive additives. Injection molding can lead to anisotropic conductivity with variability in properties, while additive manufacturing offers superior control that can result in customized and more uniform conductivity [31]. The choice of manufacturing method will depend on the project’s specifications and the required conductive properties of the final material.

This study aimed to enhance the properties of polylactic acid (PLA) by incorporating graphene nanoplatelets (GNPs) and carbon black (CB) nanoparticles. The primary objectives were to investigate the effects of these nanoparticles on the electrical conductivity, mechanical strength, and thermal behavior of PLA, particularly for applications in 3D printing and injection molding. The experiments involved the preparation of nanocomposite samples, their subsequent characterization using various analytical techniques, and a comparison of the results to determine the improvements in PLA’s performance attributable to the nanoadditives.

## 2. Materials and Methods

### 2.1. Materials

PLA commercial grade Ingeo 3850D, provided in pellet form by NatureWorks (Minnetonka, MN, USA), has a density of 1.24 g/cm^3^ and a melt flow index of 7 g/10 min. This particular grade of PLA is optimized for 3D printing, offering high strength and low crystallinity, which improve its performance and processability in printing applications.

Graphene nanoplatelets (GNPs), nanoscale plates of graphene, were supplied in powder form by US Research Nanomaterials (Houston, TX, USA). The carbon black (CB) additive, also provided by US Research Nanomaterials, Inc. (Houston, TX, USA), in powder form, consists of a mixture of superconductive carbon particles and carbon nanotubes. Polyethylene glycol (PEG), with a molecular weight of 300 g/mol as a dispersant for the nanoparticles, was purchased from Sigma-Aldrich (Schnelldorf, Germany). Its properties are detailed in Table 1.

### 2.2. Preparation of Compounding

For the preparation of the different mixtures, a twin-screw compounding monofilament extruder ZSK 18 MEGALab from Coperion (Stuttgart, Germany) was utilized, which enables the incorporation of nanomaterials into thermoplastic polymers. The formulations detailed in Table 2 were prepared to compare the injection molding process and the 3D printing process. The appropriate amounts of materials were premixed and then fed into the extruder, which operated with a temperature profile of 175 °C–175 °C–185 °C–185 °C–185 °C–195 °C–195 °C and a speed of 360 rpm.

### 2.3. Sample Manufacturing

A single-screw extruder from 3devo (Utrecht, The Netherlands) was used to produce 3D printing filaments with a diameter of 2.85 mm at an extrusion temperature of 190 °C. These filaments were then fed into a FULLMART HT 3D printer from Intamsys (Shanghai, China), operating with a hot end temperature of 205 °C and a heated bed temperature of 60 °C. The samples were manufactured with an infill pattern of 45°/−45° at 100% density and two contour lines. A 0.4 mm hot end, a 0.1 mm layer height, and a printing speed of 50 mm/s were employed. Cooling was activated at 100% from the second layer.

Injection molding of the specimens was performed in accordance with the UNE-EN ISO 527-2 standard [32]. An industrial injection molding machine, model 270/75 from Mateu-Sole (Barcelona, Spain), was used. The process temperature profile, from the feeding zone to the nozzle, was 180 °C, 185 °C, 185 °C, and 190 °C, with a filling time of 1 s and a packing time of 10 s.

### 2.4. Electrical Characterization

Surface and volume resistivity properties of the injection-molded and additive-manufactured samples were evaluated using MCP-HT450 constant voltage-supplied resistivity meters from Mitsubishi Chemical Analytech (Yamato, Japan). The average value for each sample was determined from ten measurements conducted at 25 °C.

### 2.5. Mechanical Characterization

Tensile tests were conducted using an automatic electromechanical universal testing machine, IBERTEST ELIB-50/W (S.A.E. Ibertest, Madrid, Spain), equipped with a 5 kN load cell at a speed of 5 mm/min. Samples conforming to the ISO 527-2:2012 type 1BA shape were used for these tests. The average results were calculated from five samples. Charpy impact tests were performed in compliance with ISO 179-1: 2010 [33]. Samples with a V-shaped notch, having a radius of 0.25 mm and dimensions of 80 × 10 × 4 mm, were impacted using a 1-J pendulum impact tester from Metrotec S.A. (San Sebastián, Spain). Five samples from each formulation were tested to obtain average values. Additionally, Shore D hardness was measured using a hardness tester model 637-D from Instruments J. Bot S.A. (Barcelona, Spain), following ISO 868 [34] guidelines, with a stabilization time of 15 s. Measurements were taken at five different points to obtain average data, and this process was repeated for all formulations.

### 2.6. Thermal Characterization

Thermal transitions of PLA composites were obtained by differential scanning calorimetry (DSC) in a Mettler-Toledo 821 calorimeter (Schwerzenbach, Switzerland). Samples with an average weight of 5–8 mg were subjected to a three-step program with an initial heating cycle from 25 °C to 200 °C at 10 °C min^−1^ to remove thermal history, followed by a cooling to 0 °C at −10 °C/min. Finally, a second heating cycle from 0 °C up to 350 °C at 10 °C/min was used to evaluate all thermal transitions. All tests were run in nitrogen atmosphere at a constant flow of 30 mL/min. Standard aluminum-sealed crucibles with a total volume of 40 µL were used to place the samples. In addition to above-mentioned temperatures, the degree of crystallinity (X_c_) was calculated by using the following equation (Equation (1)):

(1) $$X_{c}(\%)=\left[\frac{{∆H}_{m}-{∆H}_{cc}}{{∆H}_{m}^{0}\cdot \left(1-w\right)}\right]\cdot 100$$

where ∆H_m_ and ∆H_cc_ (J/g) correspond to the melt and cold crystallization enthalpy, respectively. ${∆H}_{m}^{0}$ (J/g) is a theoretical value that corresponds to a fully crystalline PLA. This has been taken as 93.0 J/g as reported in literature [35], and w indicates the weight fraction of total additives and/or fillers in PLA formulations.

### 2.7. Thermomechanical Characterization

Dynamic Mechanical Analysis (DMA) was conducted using a Mettler-Toledo DMA analyzer (Schwerzenbach, Switzerland) equipped with a sample holder for flexural tests. The samples, measuring 20 × 6 × 2.7 mm^3^, were analyzed over a temperature range of 30 to 140 °C with a constant temperature ramp rate of 2 °C/min. The tests were performed at a frequency of 1 Hz, with a maximum flexural deformation of 10 μm.

### 2.8. Morphological Characterization

Morphological characterization was conducted using a scanning electron microscope SEM JSM-6300 from Jeol USA Inc. (Peabody, MA, USA) with a secondary electron acceleration voltage of 15 kV. A gold coating with a layer thickness of 5–7 nm was applied to the sample surface using a sputtering process under vacuum conditions.

## 3. Results

### 3.1. Conductivity Characterization for the PLA Samples Manufactured by Additive Manufacturing and Injection Molding

**Figure 1:**
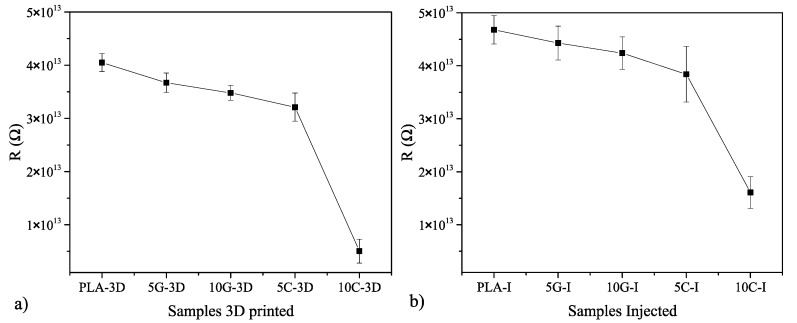
Conductivity test of PLA samples by injection and additive manufacturing in terms of resistance (R). (**a**) Conductivity of 3D printed samples. (**b**) Conductivity of injected samples.

The conductivity of the samples is measured in order to assess the addition of the nanosized particles, which are carbon-based. The resistance of the samples is measured for all the formulations proposed and the manufacturing process and is represented in Figure 1. In order to achieve the conductivity, it is necessary to achieve the percolation threshold; in the literature, in order to do this, different amounts of nanofillers are employed, but other parameters like the dispersion and the type of additive considered are also important to achieve a proper conductivity [36,37]. Nanoparticles can change PLA from 10^13^ Ω (insulative range) to the range of 10^6^ up to 10^10^ Ω (dissipative range) or even to below 10^5^ Ω (conductive region) [38]. In this work, the nanocomposites that were developed are still in the insulation range, even with the addition of 10% wt of nanoadditives. Despite being an insulator, the nanocomposites developed.

### 3.2. Mechanical Characterization for the PLA Samples Manufactured by Additive Manufacturing and Injection Molding

**Figure 2:**
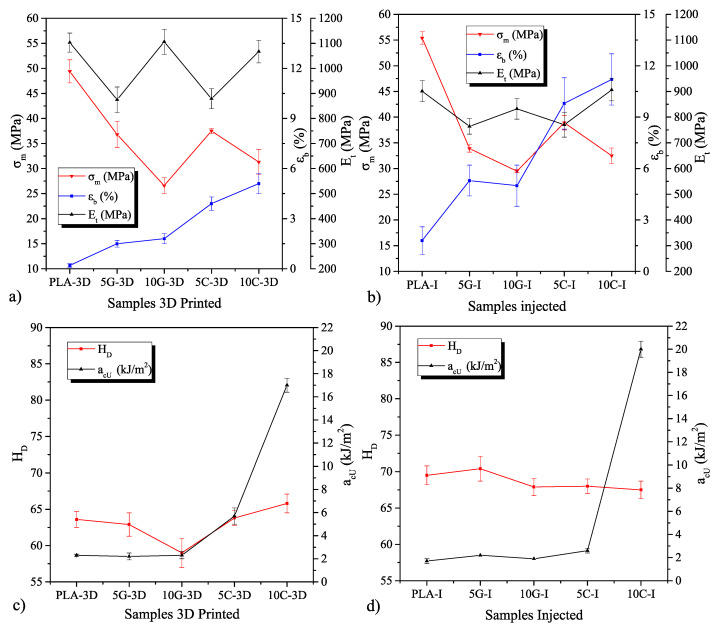
Mechanical properties in terms of maximum tensile strength (σ_m_), elongation at break (ε_b_) and tensile modulus (E_t_), shore D hardness (H_D_), and impact strength (a_cU_). (**a**) Tensile strength, elongation at break and tensile modulus of 3D printed samples. (**b**) Tensile strength, elongation at break and tensile modulus of injected samples. (**c**) Shore D hardness and impact strength of 3D printed samples. (**d**) Shore D hardness and impact strength of injected samples.

All results concerning the mechanical characterization of the materials are presented in Figure 2. The tensile behavior of the samples, depicted in Figure 2a,b, reveals changes due to the addition of nanoparticles and the manufacturing method. The tensile strength decreases with nanoparticle incorporation due to stress concentration effects commonly induced by nanoparticles. Additionally, filler dispersion plays a critical role in mechanical strength. The morphology studies indicate that carbon particles tend to agglomerate, influenced by van der Waals forces, which primarily interact with other carbon-based entities rather than with polymer chains, diminishing the reinforcement potential. A suitable strategy to enhance polymer-nanoparticle interaction is necessary to optimize reinforcement effects [39,40,41]. For neat PLA 3D-printed samples, strength values approach 50 MPa, reducing to approximately 25 MPa due to the phenomena described. Conversely, injection-molded samples exhibit slightly higher strength for the same material formulation, attributable to the orthotropic properties stemming from layer-to-layer adhesion [42,43], with a noted increase of about 5 MPa. Injection-molded samples, characterized by isotropic properties, do not experience layer-by-layer deposition. Another mechanical property assessed is the tensile modulus, which does not display a clear trend. The chain mobility of polymer samples is a key factor in stiffness; nanoparticle introduction typically reduces chain mobility, thereby enhancing stiffness. However, the effectiveness of nanoparticle interaction significantly influences stiffness enhancement [44,45]. With 5% wt of carbon-based additives, stiffness decreases, whereas a higher additive concentration approximates the stiffness to that of neat PLA. The dispersant used during manufacturing also affects stiffness, functioning as a plasticizer for PLA and enhancing ductile properties [46,47]. Prior studies have demonstrated ductility enhancements with nanofillers, even without compatibilizers [48]. Moreover, the inclusion of low-molecular-weight PEG as a dispersant, known to act as a plasticizer, significantly increases PLA elongation, with values exceeding 500% when included at 20% wt [49]. Manufacturing process variations also influence elongation at break, with additive manufacturing typically yielding lower values due to process-induced discontinuities and compromised layer adhesion [50]. The impact of plasticizers generally results in a decrease in the tensile strength of polymer materials, which is attributed to the enhancement of chain mobility [51]. In this study, as the tensile strength diminishes, the elongation correspondingly increases. Concurrently, the integration of nanoparticles with improper dispersion induces modifications in the mechanical properties, as previously suggested. The combined effects of plasticizer and nanoparticle incorporation on the developed composites contribute to the mechanical behavior observed in this research.

Figure 2c,d illustrate the impact strength and hardness of the manufactured samples. While nanofillers generally increase hardness by restricting chain mobility, the concurrent use of a plasticizing dispersant oil during manufacturing tempers this increase [52]. The combination results in minor changes in hardness values, which are also influenced by the manufacturing process. Additive manufacturing generally yields lower hardness due to orthotropic properties that vary mechanical responses based on the direction of applied force, consistently yielding values below those obtained by injection molding [53].

The impact strength of samples containing 10% wt of carbon black shows enhanced energy absorption in impact conditions. The size and shape of the nanoadditives employed significantly influence mechanical properties, as suggested by the literature, which indicates that fiber-like shapes increase impact strength by facilitating fiber debonding during material breakage [54,55]. However, it is noted that additive manufacturing reduces the energy absorption capacity, a trend also observed in comparative studies of injection molding and 3D printing processes [56].

### 3.3. Thermal Characterization for the PLA Samples Manufactured by Additive Manufacturing and Injection Molding

**Figure 3:**
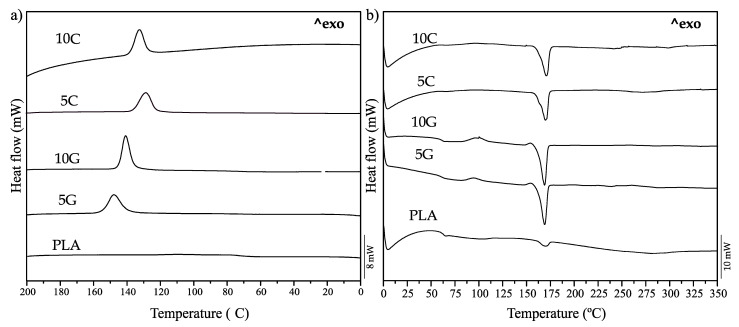
DSC thermograms of the compounded formulations employed: (**a**) cooling process and (**b**) second heating process.

Thermal analysis of PLA compounding was conducted using differential scanning calorimetry (DSC) during the second heating cycle and intermediate cooling. Figure 3 displays the thermograms from both cycles. Table 3 summarizes the primary thermal parameters associated with these DSC thermograms.

The melting temperature (T_m_) of neat PLA was found to be comparable to that of the nanocomposite variants. Notably, PLA graphene nanocomposites exhibited a reduction in this temperature, whereas those containing carbon black displayed an increase. These variations may be attributed to the distinct crystalline structures formed during crystallization, which can influence melting behavior and potentially lead to the emergence of multiple melting peaks [57]. In the research carried out by Chieng et al. [58], combining PLA/PEG/GNP in different amounts, using an X-ray diffraction (XRD) test, they show that the addition of PEG and GNP changes the PLA structure from being amorphous to having a higher crystallinity. In reference to CB, an investigation carried out by Chi-Hui Tsou et al. [59] showed by XRD test that the addition of CB to the PLA matrix increases the degree of crystallinity of PLA. By performing an XRD test, different crystal forms can be detected in PLA as α, β, and γ. Changes in the crystal form present in the polymer can be changed in different ways like the employment of nucleating agents and also by a secondary heat treatment like an annealing [60].

Nanoparticles within PLA can serve as nucleation centers for crystallization, enhancing interfacial adhesion with the PLA matrix [61,62]. The dispersion of nanocomposites has been observed to enhance crystallinity in various formulations. Furthermore, the addition of plasticizers often elevates the degree of crystallinity concurrently with an increase in elongation, attributable to enhanced chain mobility that facilitates alignment and the formation of a more compact structure [51]. In this research, the combination of the nucleating effect of the nanoparticles and the increased chain mobility collectively augmented the degree of crystallinity while also enhancing elongation capabilities. The changes observed in the degree of crystallinity are influenced by the cooling stage. As shown in Figure 3a, samples containing PEG and nanoadditives exhibited an exothermic peak during the cooling process. This indicates that the PLA composite forms crystals during cooling, a phenomenon not observed in the neat PLA sample. This effect has been observed by other authors by the addition of peroxides that act as nucleating agents, improving the low crystallization rate of neat PLA [1].

Furthermore, the cold crystallization behavior of the samples varied with the type of additive. Samples containing graphene nanoplatelets showed a minor peak, indicating different crystallization properties. Under the specified conditions, other samples managed to recrystallize completely during the cooling phase. Certain PLA grades do not fully crystallize during cooling, and alterations in composition can modify the crystallization behavior, ultimately affecting this process [1].

### 3.4. Morphological Characterization for the PLA Samples Manufactured by Additive Manufacturing and Injection Molding

**Figure 4:**
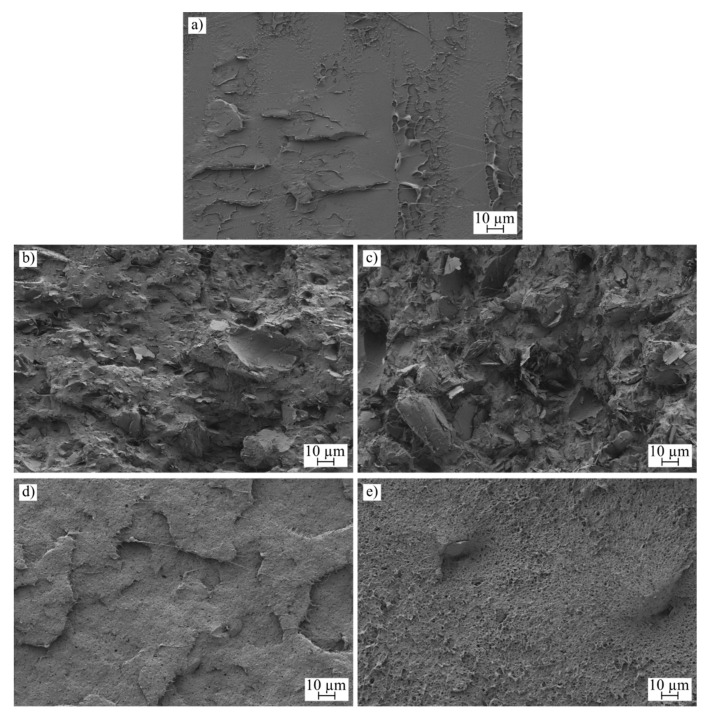
FESEM images of the injection-molded samples taken at ×500 increments: (**a**) PLA-I, (**b**) 5G-I, (**c**) 10G-I, (**d**) 5C-I, and (**e**) 10C-I.

**Figure 5:**
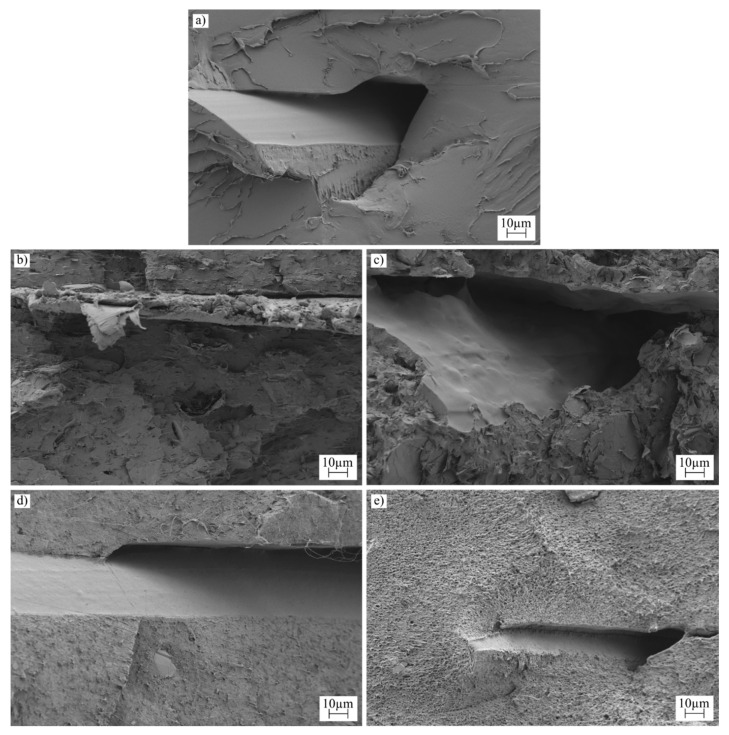
FESEM images of the 3D-printed samples taken at ×500 increments: (**a**) PLA-3D, (**b**) 5G-3D, (**c**) 10G-3D, (**d**) 5C-3D, and (**e**) 10C-3D.

**Figure 6:**
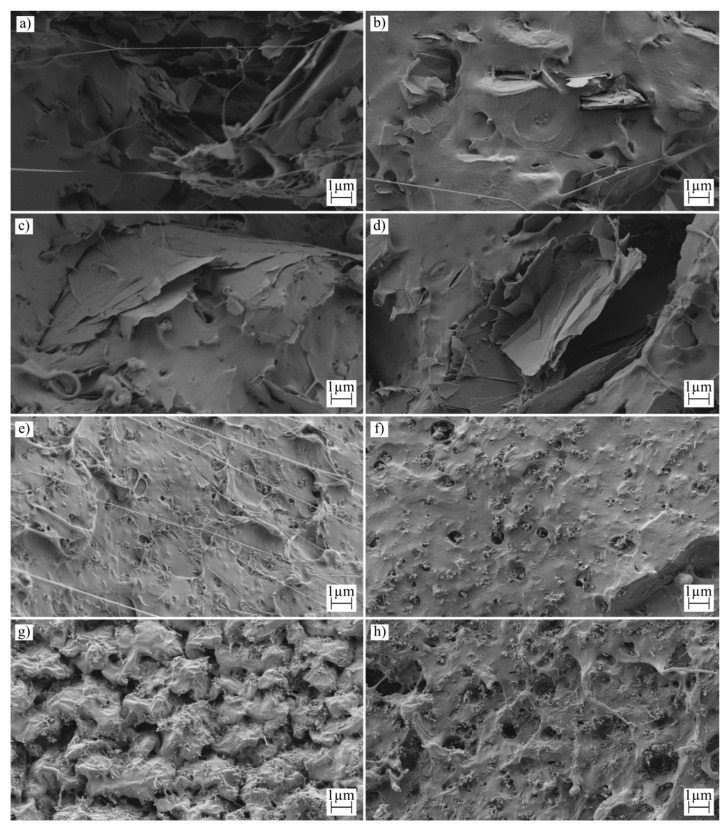
FESEM images of the 3D-printed samples taken at ×5000 increments: (**a**) 5G-3D, (**b**) 5G-I, (**c**) 10G-3D, (**d**) 10G-I, (**e**) 5C-3D, (**f**) 5C-I, (**g**) 10C-3D, and (**h**) 10C-I.

The surface morphology of all samples was analyzed using Field Scanning Electron Microscopy (FSEM), and the results are depicted in Figure 4, Figure 5 and Figure 6. Figure 4 and Figure 5 display low-magnification images, enabling observations of the sample morphology after an impact test for each manufacturing process. Additionally, Figure 6 provides a high-magnification image to facilitate the examination of nanoparticle dispersion.

Figure 4 illustrates the surface morphology of the injection-molded samples. For the neat PLA samples, the flat surface indicates a limited capacity for plastic deformation during breakage. This characteristic aligns with findings from both Charpy impact and tensile tests, where PLA is noted for its low ductility [63]. Conversely, samples containing carbon-based nanoparticles show a significantly rougher surface with visible voids, a typical outcome when powder additives are incorporated. The roughness is more pronounced in samples with graphene nanoplatelets; the flaky nature of the additive alters the surface texture, increasing roughness and causing some nanoplatelets to detach from the polymer matrix [64]. Although a slight enhancement in impact energy was noted with nanoplatelets compared to neat PLA, the weak adhesion between the filler and the polymer matrix limited further gains in energy absorption. In contrast, samples with carbon black exhibited smoother surfaces, and evidence of ductile behavior, such as filament formation during breakage, was observed [65].

Figure 5 examines samples with the same compounding, subjected to an additive manufacturing process. The formation of voids, inherent to the deposition process, is evident in this figure. These voids contribute to the development of an orthotropic behavior in the samples [66]. In this study, an infill orientation of 45/−45 degrees was used, resulting in the lines being broken during the impact test. The morphology of the rupture surface resembles that observed in the injection molding samples; however, the surfaces of the voids are smooth, a characteristic formed by the deposition process in the molten state.

The dispersion of nanoparticles was assessed at a magnification of 5000×, revealing the agglomeration of these particles, a phenomenon often observed and noted in the characterization of mechanical properties. This kind of agglomeration is widely documented in the literature [66]. Notably, variations in nanoparticle dispersion are linked to improvements in the electrical conductivity of the samples. To enhance this conductivity further, it is crucial to improve nanoparticle dispersion. Reducing agglomeration could facilitate increased percolation within the nanocomposite, potentially reaching the electrical threshold [67]. The dispersion of nanofillers varies depending on the type of additive used. Nanoplatelets, for instance, are prone to forming large agglomerates, which impacts both mechanical behavior and electrical conductivity. Across both manufacturing processes, the dispersion patterns remain consistent. However, in samples containing carbon black, a lower tendency toward agglomeration leads to improved mechanical behavior and electrical conductivity. Additionally, changes in mechanical properties related to the polymer matrix are also evident at high magnification, particularly in samples designated as 10C, which exhibit increased roughness.

### 3.5. Thermomechanical Characterization for the PLA Samples Manufactured by Additive Manufacturing and Injection Molding

**Figure 7:**
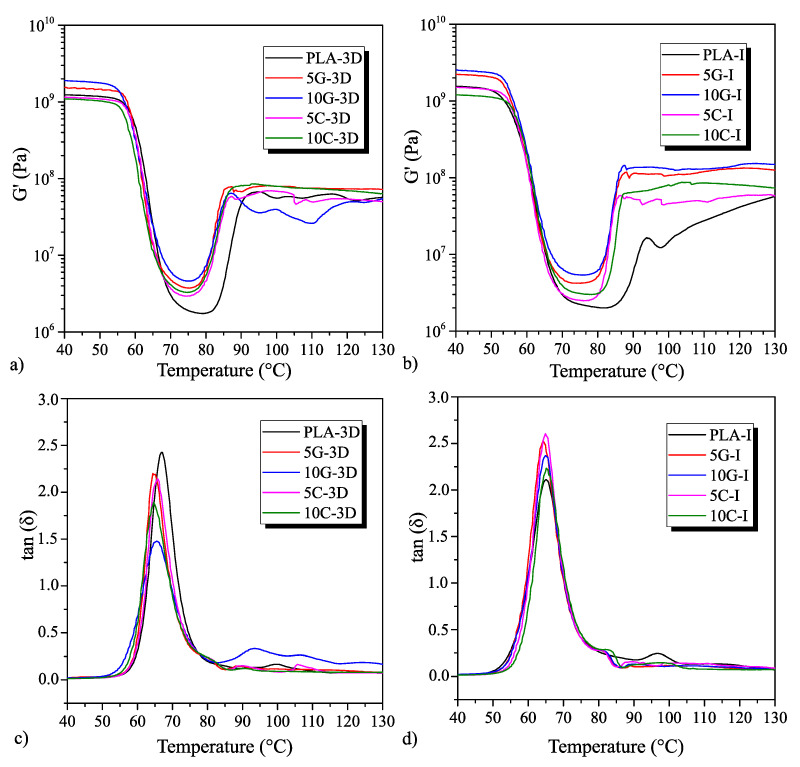
DMTA curves for the additive-manufactured and injection-molded samples in terms of storage modulus (G’) and damping factor (tan (δ)). (**a**) the G’ of the 3D printed samples. (**b**) the G’ of the injected samples. (**c**) the tan(δ) of the 3D printed samples. (**d**) the tan(δ) of the injected samples.

The thermomechanical properties of the PLA nanocomposites were analyzed using DMTA. Figure 7a,b present the storage modulus (G’) of each sample, while Figure 7c,d show the damping factor (tan(δ)).

In the initial G’ range, from 40 °C to 70 °C, both manufacturing modes show that the modulus remains unchanged despite the incorporation of nanoparticles. However, from 70 °C onwards, a significant variation in recrystallization recovery is observed, which increases in the injected specimens. The most substantial difference is shown by PLA-I, which exhibits a more progressive recrystallization, as seen in Figure 7b. Conversely, samples with both 3D-printed and injected nanoparticles show a faster recrystallization, as confirmed by DSC analysis [68].

Regarding tan(δ), the glass transition temperature (T_g_) is reduced in both manufacturing modes, as illustrated in Figure 7c,d. This reduction in T_g_ may be attributed to the addition of a compatibilizer to enhance the dispersion of the nanoparticles. Observing the tensile and impact results, it is evident that the blends tend to improve ductility and impact energy absorption. This occurs when the polymeric material is plasticized, which consequently reduces the T_g_ due to the increased mobility in the chains. The compatibilizer, therefore, produces plasticization in the blends, reducing T_g_ and increasing crystallinity [62].

Above 80 °C, a degree of recrystallization occurs under the specified heating conditions. This process, facilitated by the rearrangement of polymer chains, leads to a recovery of mechanical properties as a result of the formation of a more compact structure [69,70].

## 4. Conclusions

The comprehensive analysis detailed in this manuscript focuses on the modification of polylactic acid (PLA) using graphene nanoplatelets (GNPs) and carbon black (CB) to assess their impact on the material’s electrical conductivity, mechanical characteristics, and thermal behavior for applications in 3D printing and injection molding processes. Despite the addition of GNPs and CB, this study confirms that PLA’s electrical properties remain predominantly within the insulating range, even with nanoadditive incorporation of up to 10% by weight. This suggests that the percolation threshold necessary for significant conductivity improvement is not fully achieved under the conditions tested. Mechanical tests indicate that the inclusion of GNPs and CB generally results in a reduction of tensile strength attributable to the stress concentration effects inherent with nanoparticle integration. However, the addition of dispersants such as polyethylene glycol mitigates some of the embrittlement, enhancing the flexibility and ductility of the composite material. This improvement is crucial for applications requiring robust yet flexible materials. The isotropic properties observed in materials processed via injection molding are contrasted with the anisotropic properties found in those processed via 3D printing. Conversely, the 3D printing process, while offering design flexibility and material conservation, presents challenges in achieving consistent nanoparticle dispersion, which can affect the overall mechanical properties. Thermal analysis performed on the PLA composites reveals subtle changes in the melting behavior due to the presence of GNPs and CB, indicating an alteration in crystallization dynamics. Dynamic Mechanical Thermal Analysis (DMTA) shows a reduction of the glass transition temperature and an improvement of the recrystallization rate with the addition of nanoparticles. In conclusion, while the strategic incorporation of GNPs and CB into PLA holds significant potential for enhancing its application spectrum, the realization of these benefits is contingent upon improvements in the dispersion process of the nanoparticles. Achieving the full potential of PLA composites, however, necessitates a precise balance in the type, concentration, and dispersion of nanoparticles. This balance is essential for optimizing the material’s properties to meet specific application requirements, thus providing a pathway for the development of advanced biopolymer-based composites in both industrial and technology sectors. To address the issues highlighted, further research and refinement of processing techniques are strongly recommended to enhance nanoparticle dispersion and fully exploit the conductive and mechanical enhancements.

## Figures and Tables

**Table 1:** Main properties of the conductive additives employed for the compounding.

Property	GNP	CB
Purity (wt%)	>99	>97.5
Length (nm)	2–18	30–100
Diameter (µm)	4–12	5–30
Ph	7–7.7	8–10
Volume resistivity (Ω·cm)	4 × 10^−4^	2~5 × 10^−4^

**Table 2:** Sample name and composition (wt%) of mixtures of GNP and CB with PLA.

	Sample Name	PLA (wt%)	GNP (wt%)	CB (wt%)	PEG (phr)
Injection-molded	PLA-I	100	0	0	-
	5G-I	95	5	-	3
	10G-I	90	10	-	6
	5C-I	95	-	5	3
	10C-I	90	-	10	6
3D-printed	PLA-3D	100	0	0	-
	5G-3D	95	5	-	3
	10G-3D	90	10	-	6
	5C-3D	95	-	5	3
	10C-3D	90	-	10	6

**Table 3:** Main thermal properties extracted from the DSC test in terms of melt crystallization temperature (T_mc_), enthalpy of melt crystallization (∆H_mc_), melting temperature (T_m_), enthalpy of cold crystallization (ΔH_cc_), enthalpy of fusion (ΔH_m_), and degree of crystallinity (X_c_).

Reference	T_mc_ (°C)	∆H_mc_ (J/g)	T_m_ (°C)	∆H_cc_ (J/g)	∆H_m_ (J/g)	X_c_ (%)
PLA	-	-	169.2 ± 1.5	-	7.1 ± 0.3	7.6 ± 0.3
5G	147.3 ± 0.8	35.3 ± 1.4	168.5 ± 1.2	2.7 ± 0.2	38.9 ± 1.4	41.0 ± 0.8
10G	140.3 ± 0.5	37.2 ± 1.5	168.4 ± 0.9	7.6 ± 0.3	40.8 ± 1.3	39.7 ± 0.7
5C	129.5 ± 0.9	33.2 ± 1.3	169.9 ± 1.2	-	34.0 ± 1.2	38.5 ± 0.8
10C	133.7 ± 0.8	30.2 ± 1.2	170.6 ± 1.1	-	33.5 ± 1.4	40.0 ± 0.8

## Data Availability

The original contributions presented in the study are included in the article, further inquiries can be directed to the corresponding author.
